# Association between ABO/Rh blood groups and transfusion-transmitted infections among Turkish blood donors: a comprehensive demographic analysis (2015–2021)

**DOI:** 10.1186/s12879-026-12842-5

**Published:** 2026-02-13

**Authors:** Aziz Karaca, Özgür Rüştü Güner, Mustafa Nuri Günçıkan, Mustafa Yılmaz, Gizem Gökçe Karadağ, Nazlı Nadire Sözmen, Levent Sağdur, Kerem Kınık, Fatma Meriç Yılmaz

**Affiliations:** 1 Faculty of Medicine, Department of Physiology, Recep Tayyip Erdoğan University, Rize, Türkiye; 2 Turkish Red Crescent, General Directorate of Blood Services, Ankara, Türkiye; 3 General Directorate of Blood Services, Infectious Diseases and Clinical Microbiology Unit, Turkish Red Crescent, Ankara, Türkiye; 4 Turkish Red Crescent, Biostatistics Unit, General Directorate of Blood Services, Ankara, Türkiye; 5 Department of Emergency Management and Disaster, University of Health Sciences, Istanbul, Türkiye; 6 Turkish Red Crescent, Managing Board, Ankara, Türkiye; 7 Faculty of Medicine, Department of Biochemistry, Yıldırım Beyazıt University, Ankara, Türkiye

**Keywords:** ABO and Rh blood groups, Blood transfusion, Demographic characteristics, Transfusion-transmitted infections, Turkish blood donor, Turkish Red Crescent

## Abstract

**Background:**

This study assessed associations between transfusion-transmitted infections (TTIs: hepatitis B, hepatitis C, HIV, syphilis) and demographic factors (sex, age, education) including ABO/Rh blood groups among Turkish blood donors from 2015 to 2021.

**Methods:**

A retrospective cross-sectional analysis was performed on 56,766 donors confirmed with any TTI and permanently deferred from donation. The control group comprised 7,229,867 volunteer donors from the same period. Multivariable logistic regression analysis was conducted to assess independent associations between demographic factors and ABO/Rh blood groups with individual TTI risks, adjusting for potential confounders. False Discovery Rate correction was applied for multiple comparisons.

**Results:**

Overall TTI prevalence was 0.779%, with HBV being most prevalent (0.594%), followed by syphilis (0.133%), HCV (0.031%), and HIV (0.024%). TTI prevalence decreased significantly over the study period. Male donors showed higher adjusted risk for all TTIs. Lower education levels (≤ 8 years) were associated with increased risk for all infections. Younger donors (18–34 years) had lower prevalence of HBV and syphilis compared to those ≥ 35 years. After FDR correction, several blood group associations remained significant: Blood group B Rh(+) showed increased risk for both HBV (aOR = 1.059, 95% CI: 1.032–1.088) and syphilis (aOR = 1.121, 95% CI: 1.062–1.184). AB Rh(+) donors had increased syphilis risk (aOR = 1.118, 95% CI: 1.035–1.206). Conversely, O Rh(+) showed protective association with syphilis (aOR = 0.855, 95% CI: 0.817–0.895), while A Rh(-) demonstrated protection against HBV (aOR = 0.935, 95% CI: 0.894–0.978). No significant associations were found between blood groups and HCV or HIV infections.

**Conclusion:**

This large-scale study demonstrates declining TTI prevalence in Turkish blood donors and identifies demographic risk factors including male sex, older age, and lower education. ABO/Rh blood groups show statistically significant but modest associations with specific TTIs, particularly HBV and syphilis. These findings suggest blood group systems may represent non-modifiable risk factors through complex immunological mechanisms. Comprehensive molecular and genetic studies are needed to elucidate underlying mechanisms and validate these population-specific associations.

**Clinical trial:**

Not applicable.

**Supplementary Information:**

The online version contains supplementary material available at 10.1186/s12879-026-12842-5.

## Background

Blood transfusion is a life-saving medical procedure, and blood components are listed among the World Health Organization (WHO) essential medicines [1]. However, unsafe transfusion can result in transfusion-transmitted infections (TTIs), primarily caused by human immunodeficiency virus (HIV), hepatitis B virus (HBV), hepatitis C virus (HCV), and Treponema pallidum (syphilis). WHO recommends universal screening of all blood donations for these pathogens to minimize the risk of fatal or chronic complications. Despite these measures, TTIs remain a public health concern, with prevalence varying widely across regions and countries [2].

In addition to behavioral and environmental risk factors, host genetic factors such as ABO and Rh blood group antigens may influence susceptibility to infections [3]. Currently, the International Society of Blood Transfusion (ISBT) reports that there are a total of 47 blood group systems containing 366 antigens on the surface of erythrocytes [4]. Although many blood group systems have been defined, their immunogenicity in blood transfusions, especially ABO and Rh blood group systems, has been a focus. In humans, ABO antigens expressed on tissues such as epithelial and vascular endothelial cells as well as erythrocytes have been associated with a wide variety of diseases [5]. Rh (D) phenotypes have also been associated with several diseases [6]. Previous studies have reported conflicting results regarding the association between ABO/Rh blood groups and TTIs, which may be due to differences in study design, sample size, adjustment for confounders, or population characteristics [7, 8].

In Türkiye, limited research has examined both the epidemiology of TTIs and their possible association with ABO/Rh blood groups in blood donors, and existing findings are inconsistent [9]. This highlights the need for large-scale, methodologically robust studies. Therefore, the present study aimed to: (1) assess TTI prevalence trends among Turkish blood donors between 2015 and 2021, (2) identify demographic risk factors, and (3) evaluate the independent association between ABO/Rh blood groups and individual TTI risks after adjusting for potential confounders.

## Methods

### Study design and population

This retrospective cross-sectional study included 56,766 blood donors from the Turkish Red Crescent (TRC) who were confirmed to have any transfusion-transmissible infections (TTIs) and who permanently deferred between January 2015 and December 2021. The control group consisted of 7,229,867 volunteer blood donors from the same period. To determine the actual blood group distribution, only the last donation of each donor was considered. To achieve a relatively balanced age distribution, the donors were categorized into two age groups: 18–34 years and ≥ 35 years, following our previous study [10]. Educational status was classified as ≤ 8 years education (illiterate, primary school, middle school, high school) and ≥ 9 years education (associate degree, undergraduate, graduate, doctorate).

### Blood donation criteria and testing

The TRC supplies approximately 90–91% of the country’s blood and blood components. Before donation, all blood donors are required to complete the standardized blood donor screening questionnaire and informed consent forms as specified in the Turkish Ministry of Health’s ‘’National Blood and Blood Components Preparation, Use and Quality Assurance Guidelines 2016‘’ [11]. These forms represent the mandatory national standard used by all blood centers in Türkiye. Blood donors must be between 18 and 70 years old, weigh over 50 kg, have a hemoglobin level of at least 12.5 g/dL in women and 13.5 g/dL in men, and have a normal heart rate between 50 and 100 beats per minute. The systolic blood pressure of these patients should be between 90 and 180 mmHg, whereas the diastolic blood pressure should range from 60 to 100 mmHg. Additionally, they should have no prior history of any transfusion-transmissible infection. The TRC conducts serological tests on blood donations, and since October 30, 2014, additional nucleic acid amplification testing (NAT) has been implemented.

### Blood group determination and TTI screening protocols

Since 2015, blood groups have been determined in immunohaematology laboratories via two parallel methods. The first method is the gel centrifuge technique, which is performed with the Grifols-Erytra (Grifols, Spain) device and Grifols kits. The second method is the microplate technique, which is performed via the Beckman Coulter PK 7400 (Beckman Coulter, Japan) device and Diagast (Beckman Coulter, France) kits.

On the other hand, all TTI screening protocols described in this study were uniformly standardized and applied across all TRC blood collection centers nationwide, which supply approximately 90–91% of Türkiye’s blood and blood components. Supplemental Table 1 provides further details regarding the screening and confirmatory test methods used.

### Donor deferral and testing workflow

Supplemental Fig. 1 illustrates the decision mechanisms related to blood component eligibility on the basis of screening test results. Donors are temporarily deferred until confirmatory testing is completed. If a confirmatory test returns positive, the donor is permanently deferred.

### Ethical approval and data collection

This study was approved by TRC Research Ethics Committee (Approval number: 2022/05; 10.05.2022). All methods were performed in accordance with the relevant guidelines and regulations, including the Declaration of Helsinki. Donor data were retrieved from the TRC database.

### Statistical analysis

To assess the independent associations between **ABO/Rh blood groups (predictor variables)** and the risk of each TTI (HBV, HCV, HIV, syphilis), multivariable logistic regression analysis was conducted. This approach allowed for the simultaneous adjustment of potential confounding variables—**specifically sex**,** age group**,** and education level**—enabling a more robust evaluation of independent risk factors.

**Four separate multivariable logistic regression models were fitted**,** one for each TTI type (HBV**,** HCV**,** and Syphilis) as the binary outcome.**

Models were adjusted for:

1. **Sex** (Reference: Female).
2. **Education level** (≤ 8 vs. ≥ 8 years; Reference: >8 years).
3. **Age group** (18–34 vs. ≥ 35 years; Reference: 18–34 years).

The decision to categorize age, rather than using it as a continuous variable, was made to account for potential **non-linear relationships** between age and TTI risk (e.g., risk thresholds) and to provide more **clinically interpretable risk thresholds**. The cut-point of 35 years was specifically chosen as it yielded **comparable group sizes** within our donor population, enhancing the statistical stability and power of the comparison.

We applied False Discovery Rate (FDR) correction using the Benjamini-Hochberg method to account for multiple comparisons. Given the very large sample size, it is well recognized that even very small differences may yield statistically significant p-values [12]. Therefore, in interpreting the results, emphasis was placed not solely on p-values, but on the magnitude of Adjusted Odds Ratio (aOR) and their 95% Confidence Intervals (CIs) as indicators of effect size and practical significance.

**The predictor variables**,** ABO/Rh groups**,** were defined** such that each specific group (e.g., “A Rh+”) was compared against all other blood groups combined (Reference: All other groups). Statistical analyses were performed using R software (version 4.5.2, R Foundation for Statistical Computing, Vienna, Austria), and a two-tailed p-value of less than FDR-adjusted 0.05 was considered statistically significant.

## Results

### Demographic characteristics

The mean age of TTI-positive donors included in the study was 41.7 ± 12.4 years, whereas the mean age of the control group was 34.8 ± 11.3 years. Regarding sex distribution, 89.7% (*n* = 50,927) of donors in the TTI group were male and 10.3% (*n* = 5,839) were female, compared to 81.6% (*n* = 5,900,687) male and 18.4% (*n* = 1,329,180) female in the control group. The distribution by educational level for both TTI-positive and control donors is detailed in Table 1.

**Table 1:** Demographic characteristics of TTI and control groups (2015–2021)

Demographic Characteristic	TTI Group (*n* = 56,766)	Control Group (*n* = 7,229,867)
**Age (years**,** mean ± SD)**	41.7 ± 12.4	34.8 ± 11.3
**Sex (n**,** %)**		
Male	50,927 (89.7%)	5,900,687 (81.6%)
Female	5,839 (10.3%)	1,329,180 (18.4%)
**Education Level (n**,** %)**		
Literate	1,250 (2.3%)	67,541 (0.9%)
Primary School	17,903 (32.5%)	1,211,579 (17.1%)
Middle School	9,727 (17.6%)	942,237 (13.3%)
High School	15,701 (28.5%)	2,266,618 (32.0%)
Associate Degree	2,959 (5.4%)	766,863 (10.8%)
Bachelor’s Degree	6,478 (11.7%)	1,627,019 (22.9%)
Master’s Degree	847 (1.5%)	162,929 (2.3%)
Doctorate	296 (0.5%)	36,505 (0.5%)

### Blood group distribution

**Fig. 1:**
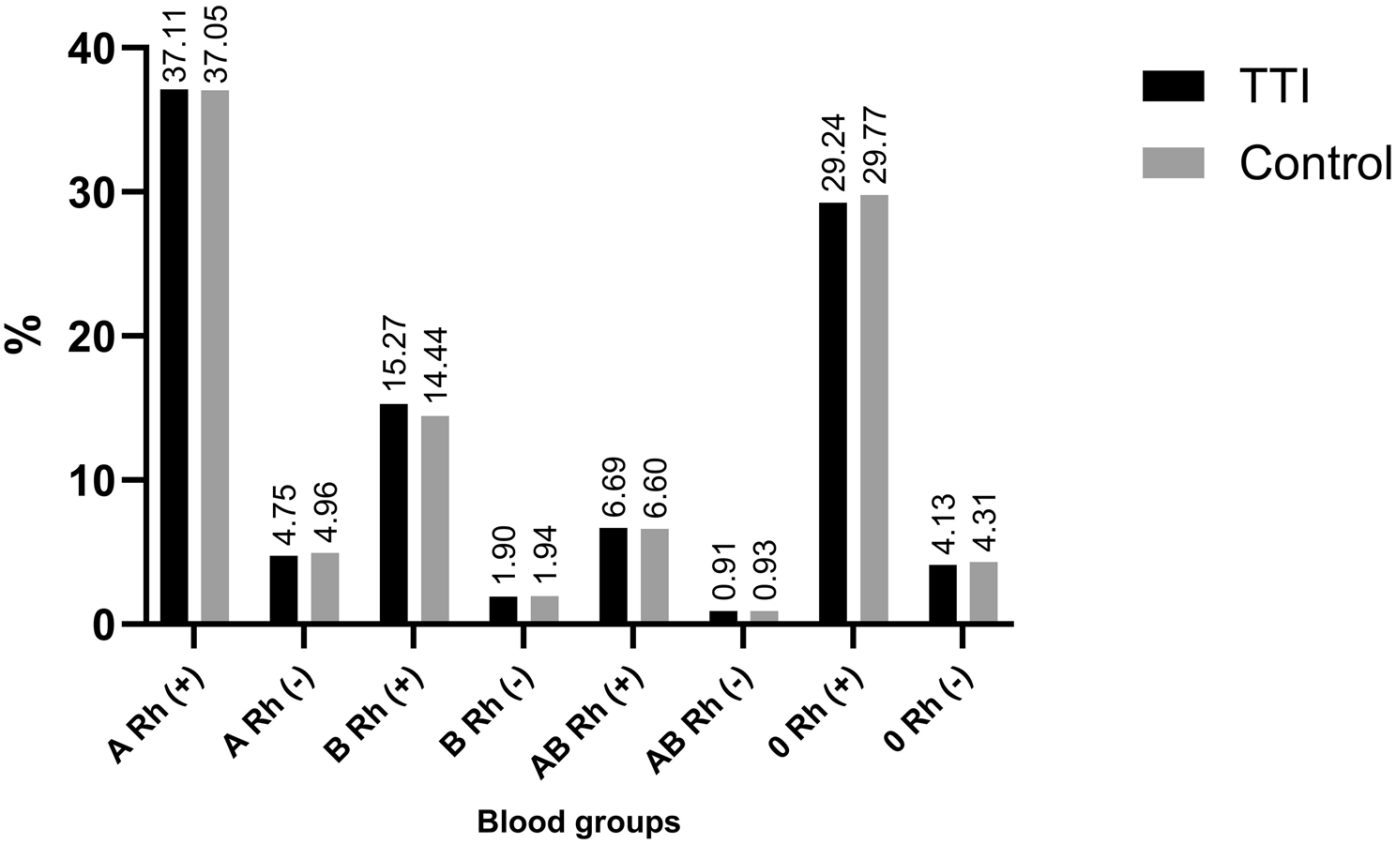
ABO and Rh blood group distributions in the TTI and control groups

The distributions of ABO and Rh blood groups showed similar overall patterns in both groups. In both the TTI and control groups, A Rh (+) was the most common blood type, followed by O Rh (+). The rarest blood group was AB Rh (-), followed by B Rh (-) (Fig. 1).

### Prevalence of transfusion-transmissible infections (TTIs)

**Fig. 2:**
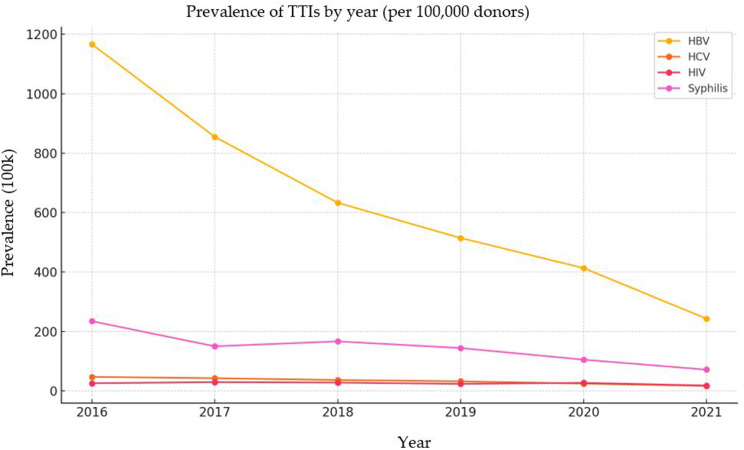
HBV, HCV, HIV, and Syphilis course by years

Overall TTI prevalence was 0.779%, with HBV being most prevalent (0.594%), followed by syphilis (0.133%), HCV (0.031%), and HIV (0.024%). There was a significant decrease in the prevalence of all TTI agents over the years (Fig. 2).

### Temporal trends in TTI prevalence

All TTIs demonstrated statistically significant declining trends during the study period (Table 2; Fig. 2). Among TTI-positive donors, HBV prevalence decreased from 1.44% (95% CI: 1.41–1.47) in 2015 to 0.24% (95% CI: 0.23–0.25) in 2021 (Extended Mantel-Haenszel test, *p* < 0.001), representing an 83.3% relative reduction. Similarly, HCV declined from 0.054% to 0.017% (68.5% reduction, *p* < 0.001), HIV from 0.023% to 0.018% (21.7% reduction, *p* < 0.001), and syphilis from 0.21% to 0.07% (66.7% reduction, *p* < 0.001).

**Table 2:** Annual prevalence of individual TTIs among TTI-positive blood donors (2015–2021)

Year	HBV (+) *n*	HBV (-) *n*	% (95% CI)	HCV (+) *n*	HCV (-) *n*	% (95% CI)	HIV (+) *n*	HIV (-) *n*	% (95% CI)	Syphilis (+) *n*	Syphilis (-) *n*	% (95% CI)
2015	7490	1545	1.44 (1.41–1.47)	286	8749	0.054 (0.047–0.063)	121	8914	0.023 (0.019–0.027)	1138	7897	0.21 (0.20–0.23)
2016	7210	1902	1.17 (1.14–1.19)	293	8819	0.047 (0.041–0.052)	160	8952	0.026 (0.022–0.029)	1449	7663	0.23 (0.22–0.24)
2017	6399	1666	0.85 (0.83–0.88)	320	7745	0.042 (0.038–0.047)	222	7843	0.029 (0.026–0.033)	1124	6941	0.15 (0.14–0.16)
2018	5731	2098	0.63 (0.62–0.65)	333	7496	0.036 (0.033–0.040)	255	7574	0.028 (0.025–0.032)	1510	6319	0.17 (0.16–0.18)
2019	6454	2513	0.51 (0.50–0.53)	406	8561	0.032 (0.029–0.035)	297	8561	0.024 (0.021–0.026)	1810	7157	0.14 (0.13–0.15)
2020	4932	1871	0.41 (0.40–0.43)	293	6510	0.024 (0.022–0.027)	325	6478	0.027 (0.024–0.030)	1253	5550	0.10 (0.09–0.11)
2021	4837	2118	0.24 (0.23–0.25)	336	6619	0.017 (0.015–0.019)	359	6619	0.018 (0.016–0.020)	1423	5532	0.07 (0.06–0.08)
	56,766*			323.05*			16.52*			1330.22*		
	**p-value < 0.001**			**p-value < 0.001**			**p-value < 0.001**			**p-value < 0.001**		

* Extended Mantel-Haenszel chi square for linear trend

CI: Confidence Interval; HBV: Hepatitis B Virus; HCV: Hepatitis C Virus; HIV: Human Immunodeficiency Virus

### Adjusted associations between demographic factors and TTIs

The results of the multivariable logistic regression analysis revealed independent associations between demographic factors and the risk of TTIs. By adjusting for other confounding variables, these analyses allowed for the estimation of the true contribution of each factor to TTI risk. Due to the very large sample size, many associations were found to be statistically significant (*p* < 0.05); however, it is essential to interpret these findings in conjunction with their practical significance. The magnitude of the odds ratios (ORs) and the corresponding 95% confidence intervals provide more meaningful insight into the effect sizes [12]. The adjusted associations between demographic factors and TTIs are summarized in Table 3.

**Table 3:** Adjusted odds ratios and 95% confidence intervals for associations between demographic Factors, ABO/Rh blood Groups, and Transfusion-Transmitted infections: multivariable logistic regression analysis

Variable	HBV positive cases: 43,053		HCV positive cases: 2,267		HIV positive cases: 1,739		Syphilis positive cases: 9,707	
	aOR (95% CI)	FDR *p*	aOR (95% CI)	FDR *p*	aOR (95% CI)	FDR *p*	aOR (95% CI)	FDR *p*
A Rh (+) vs. Others	0.991 (0.972-1.011)	0.573	0.927 (0.85-1.01)	0.168	1.088 (0.988-1.198)	0.171	1.045 (1.003-1.089)	0.084
A Rh (-) vs. Others	0.935 (0.894-0.978)	**<0.05**	0.998 (0.825-1.206)	0.981	0.899 (0.716-1.128)	0.572	1.022 (0.934-1.119)	0.778
B Rh (+) vs. Others	1.059 (1.032-1.088)	**< 0.001**	1.118 (0.999-1.251)	0.116	0.931 (0.811-1.068)	0.540	1.121 (1.062-1.184)	**<0.001**
B Rh (-) vs. Others	0.967 (0.902-1.037)	0.574	0.958 (0.706-1.3)	0.848	1.047 (0.749-1.464)	0.848	1.025 (0.889-1.182)	0.841
AB Rh (+) vs. Others	0.988 (0.951-1.027)	0.709	1.056 (0.898-1.241)	0.702	0.963 (0.794-1.167)	0.816	1.118 (1.035-1.206)	**<0.05**
AB Rh (-) vs. Others	0.978 (0.885-1.081)	0.793	0.956 (0.615-1.484)	0.884	1.121 (0.704-1.783)	0.778	0.935 (0.755-1.158)	0.708
O Rh (+) vs. Others	1.003 (0.982-1.024)	0.848	1.047 (0.957-1.145)	0.540	0.990 (0.893-1.098)	0.884	0.855 (0.817-0.895)	**<0.001**
O Rh (-) vs. Others	0.972 (0.927–1.019)	0.447	0.795 (0.634–0.996)	0.105	0.901 (0.706–1.151)	0.618	0.962 (0.871–1.063)	0.663
Sex (Male vs. Female)	1.778 (1.722-1.836)	**< 0.001**	1.193 (1.06-1.342)	**< 0.05**	6.261 (4.817-8.138)	**< 0.001**	1.263 (1.19-1.34)	**< 0.001**
Age (18-34 vs ≥35 years)	0.449 (0.440-0.459)	**< 0.001**	1.058 (0.973-1.149)	0.355	0.900 (0.818-0.99)	0.074	0.412 (0.394-0.43)	**< 0.001**
Education (≤ 8 vs. ≥ 9 years)	2.174 (2.120-2.228)	**< 0.001**	2.982 (2.663-3.339)	**< 0.001**	1.178 (1.063-1.306)	**<0.05**	2.070 (1.967-2.18)	**< 0.001**

Note: All models adjusted for sex, age, education, and blood groups with FDR correction

aOR: Adjusted Odds Ratio; CI: Confidence Interval; FDR: False Discovery Rate; HBV: Hepatitis B Virus; HCV: Hepatitis C Virus; HIV: Human Immunodeficiency Virus

**Fig. 3:**
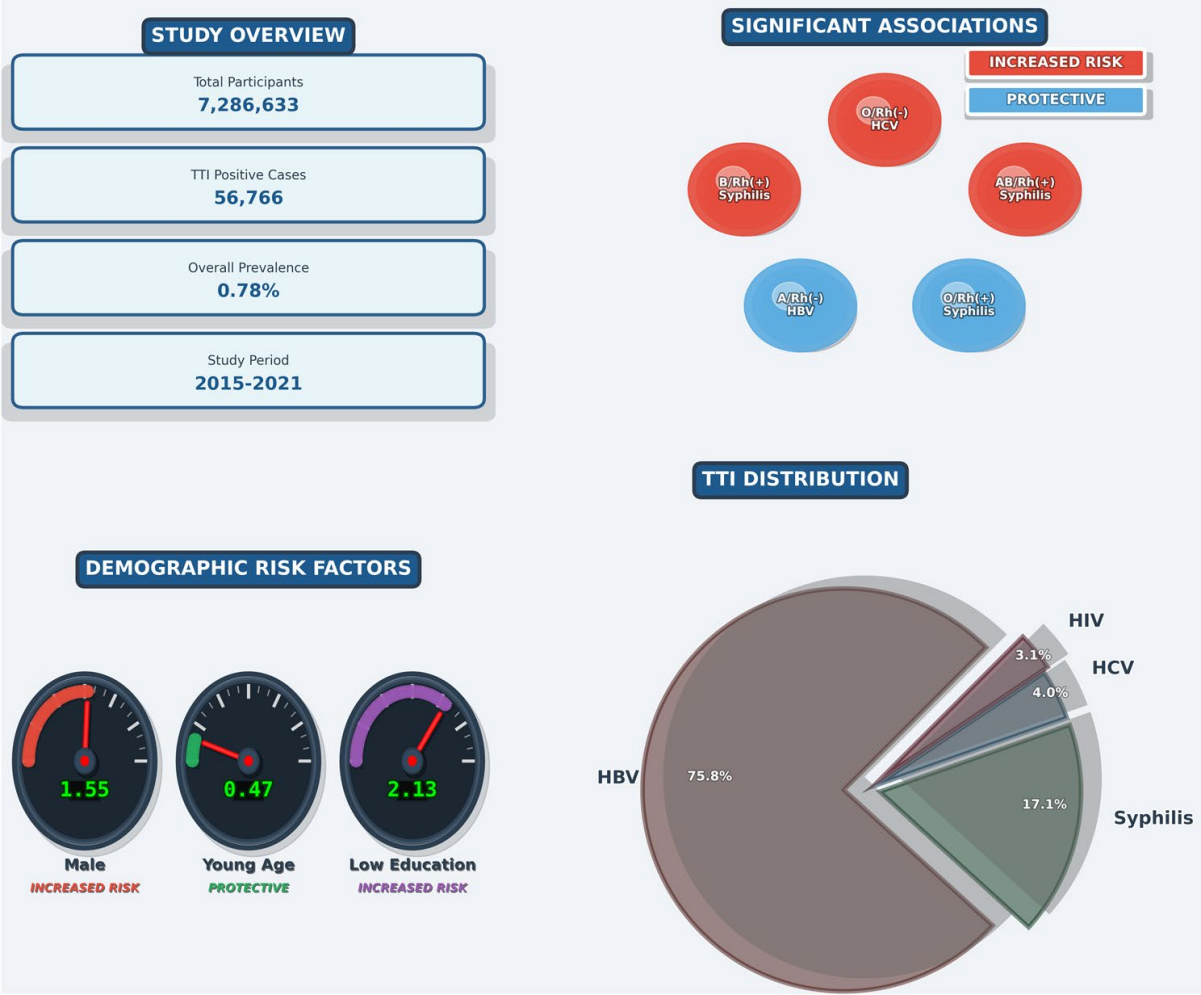
Study overview: population characteristics, risk factors, and key findings

Male donors were found to have a higher adjusted risk for all TTIs. Similiarly, in the low education group (≤ 8 years), there was a significant increase in the risk of all TTIs compared to the high education group (≥ 9 years). On the other hands, compared to donors aged 35 and over, those in the 18–34 age group showed a lower adjusted prevalence of HBV and syphilisis (Table 3; Fig. 3).

### Adjusted associations between blood groups and TTIs

**Fig. 4:**
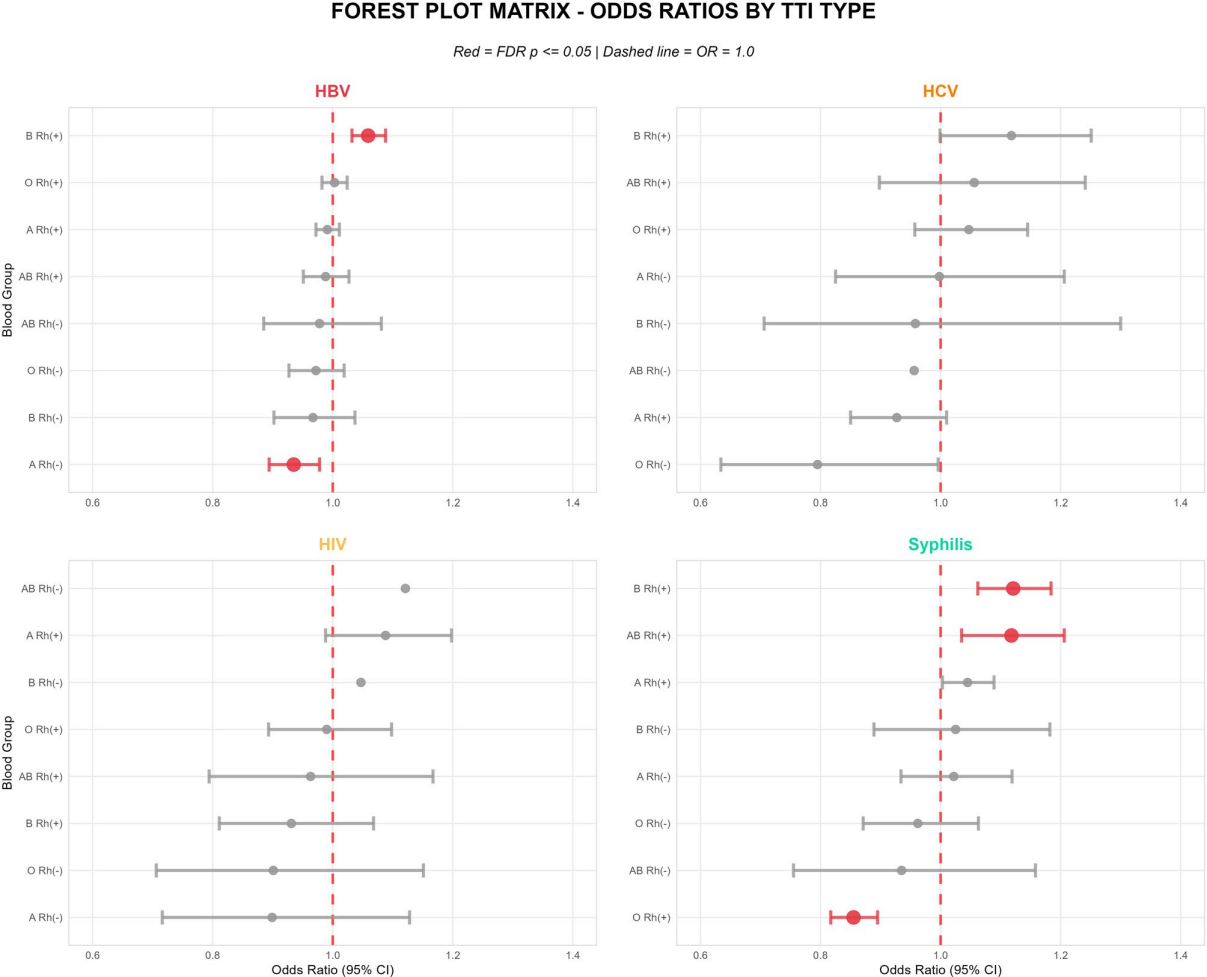
Forest plot showing adjusted odds ratios and 95% confidence intervals for associations between ABO/Rh blood groups and transfusion-transmitted infections after multivariable logistic regression analysis

When examining the relationship between blood groups and TTIs, some independent associations were observed even after adjusting for demographic factors. The adjusted risk associations between blood groups and TTIs are summarized in Table 3. After FDR correction, several blood group associations remained statistically significant. Blood group B Rh (+) showed increased risk HBV (aOR = 1.059, 95% CI: 1.032–1.088, *p* < 0.001) and syphilis (aOR = 1.121, 95% CI: 1.062–1.184, *p* < 0.001). Similarly, blood group AB Rh (+) demonstrated increased syphilis risk (aOR = 1.118, 95% CI: 1.035–1.206, *p* = 0.012). Conversely, protective associations were observed for blood group O Rh (+) with syphilis (aOR = 0.855, 95% CI: 0.817–0.895, *p* < 0.001) and for blood group A Rh (-) with HBV (aOR = 0.935, 95% CI: 0.894–0.978, *p* < 0.05) (Fig. 4).

## Discussion

This large-scale study of 56,766 TTI-positive donors and over 7 million controls from the Turkish Red Crescent (2015–2021) reveals several principal findings. First, TTI prevalence declined substantially across all pathogens during the study period, with HBV showing the most dramatic reduction (83.3%). Second, demographic risk factors including male sex, older age (≥ 35 years), and lower education (≤ 8 years) were independently associated with increased TTI risk across multiple infection types. Third, specific ABO/Rh blood group phenotypes demonstrated modest but statistically significant associations with HBV and syphilis infections after adjustment for demographic confounders, suggesting potential non-modifiable biological risk factors that warrant further investigation. These findings have implications for blood safety policy, targeted donor education strategies, and future research directions in Türkiye and comparable settings.

In this context, large-scale meta-analyses have demonstrated that non-O blood groups may show increased susceptibility to various infections, while demographic factors can create substantial differences in infection risk across age, sex, and socioeconomic groups [13]. The molecular basis of these relationships may potentially involve direct pathogen-host interactions mediated by blood group antigens, natural antibody neutralization mechanisms, and demographically specific variations in immune function, though the precise mechanisms remain speculative and require further investigation. Understanding these relationships is critical as global blood safety initiatives expand across diverse populations with varying demographic profiles, genetic backgrounds, and pathogen exposure histories.

### Prevalence and temporal trends of TTIs

The overall TTI prevalence of 0.779% positions Türkiye as an intermediate-endemic country in the global blood safety landscape. HBV (0.594%) was the most prevalent infection, followed by syphilis (0.133%), HCV (0.031%), and HIV (0.024%). When compared with neighboring countries in the Middle East and Eastern Mediterranean region, our HBV prevalence was moderately higher than Iran (0.39%) [14], Jordan (0.38%) [7], the UAE (0.234%) [15], and Iraq (0.6%) [16]. However, our HCV prevalence (0.031%) was notably lower than these countries (ranging from 0.11% to 0.3%) [7, 14–16], which may reflect the cumulative impact of direct-acting antiviral treatment programs and harm reduction initiatives implemented in Türkiye over the past decade [17]. Our HIV (0.024%) and syphilis (0.133%) rates were higher than those reported in Iran (0.005%, 0.01%) and Jordan (0.006%, 0.02%) [7, 16], which can likely be attributed to differences in screening protocols, demographic composition, and sexually transmitted infection epidemiology. Regional differences may also reflect the timing of HBV vaccination program initiation (Iran 1993 [18] vs. Türkiye 1998 [19]), NAT implementation dates, migration dynamics, and socioeconomic determinants.

In the broader global context, high-income countries such as Germany report substantially lower rates (HBV 0.12–0.14%, HCV 0.07–0.08%) [20], and the Netherlands demonstrates TTI prevalence among blood donors 6–60 times lower than in the general population [21]. In contrast, sub-Saharan African countries exhibit dramatically higher overall TTI prevalence, including Uganda (8.7%) [22], Ethiopia (7.4%) [23], and Nigeria (8.3%) [24]. These comparisons underscore Türkiye’s intermediate position in the global blood safety landscape and its progressive trajectory toward developed-nation standards.

One of the most significant findings of our study is the statistically significant declining trend observed across all TTIs between 2015 and 2021. HBV prevalence decreased from 1.44% to 0.24%, representing an 83.3% reduction (*p* < 0.001). Similarly, significant declines were observed in syphilis (66.7%), HCV (68.5%), and HIV (21.7%) (all *p* < 0.001). Year-by-year analysis revealed distinct temporal dynamics. HBV showed the steepest decline between 2017 and 2019, coinciding with nationwide expansion of NAT coverage to all blood centers, followed by more gradual reduction through 2021 as the donor pool “cleaning effect” reached saturation. HCV demonstrated accelerated decline post-2018, corresponding to widespread DAA therapy availability under universal health coverage. HIV prevalence remained relatively stable until 2019, then declined in 2020–2021, potentially reflecting enhanced pre-donation screening and targeted prevention campaigns. Syphilis showed consistent linear decline throughout the study period, indicating sustained effectiveness of serological screening. These declining trends align with reductions reported in other middle-income countries [22, 23], suggesting that investments in blood safety infrastructure yield measurable epidemiological improvements.

This decline reflects the synergistic effect of multifactorial interventions. NAT implementation in 2014 shortened diagnostic window periods to approximately 20 days for HBV and 7 days for HCV [25], enabling earlier detection of infected donors and progressively “cleaning” the donor pool. This effect is compounded by the high proportion of regular donors (53% in 2021) [26], indicating a mature, well-screened donor population. The HBV vaccination program initiated in 1998 [19] created a cohort effect, with all donors aged 18–23 years being vaccinated by 2021. Enhanced screening protocols implemented in 2016 [11] and widespread availability of DAA therapies achieving greater than 95% cure rates for HCV further contributed to reduced community burden of undiagnosed infections. The expansion of universal health insurance coverage has also improved access to treatment and reduced undiagnosed infections in the general population. Sustaining this progress toward WHO’s 2030 hepatitis elimination targets will require continued vigilance, maintenance of high NAT coverage, and uninterrupted vaccine supply.

### Sex-related patterns for TTIs

Male donors demonstrated higher adjusted risk for all TTIs in our study. This finding is consistent with previous research conducted in Türkiye showing significant association between male sex and HBsAg positivity (OR 1.77; 95% CI 1.28–2.46; *p* < 0.001) [27]. Similarly, international studies have reported higher TTI prevalence among male donors, with Gebreyes et al. noting elevated HBV and syphilis rates in men [23], Aliyo and colleagues observing higher overall TTI prevalence in males with specifically elevated syphilis and HBV rates [28], and Deshmukh et al. finding higher HBV, HCV, and HIV prevalence in male donors [29]. Brown et al. described sex dimorphism in HBV infection, characterized by greater exposure to infection and more frequent complications such as cirrhosis and hepatocellular carcinoma in males. These differences have been explained by differential immune responses between sexes, sex dimorphism in liver physiology, and the presence of androgen response elements in the HBV genome [30]. The higher risk of infections such as HIV in men has been attributed in part to behavioral factors, including tendency to engage in higher-risk sexual practices [31].

Several biological mechanisms have been proposed that might contribute to sex differences in infection susceptibility, though these remain speculative in the context of our observational data. Testosterone may generally suppress immune responses by binding to androgen response elements in host cell genomes, reducing antibody production and cytotoxic T cell responses. Conversely, estrogen may enhance immune function through increased immune cell activation and stronger antibody responses, potentially leading to better vaccine-induced immunity and protection against infections in women [32, 33]. X-chromosome gene escape, whereby approximately 15% of X-linked immune genes including TLR7, CD40L, and FOXP3 escape inactivation in females, might provide additional immune advantages. TLR7 escape has been associated with enhanced interferon-α production in approximately 30% of female immune cells, contributing to stronger antiviral responses [34]. However, given the cross-sectional observational design of our study, we cannot determine the relative contributions of behavioral versus biological factors to the observed sex differences, and these mechanistic hypotheses require validation in dedicated immunological studies.

### Age-related patterns for TTIs

The prevalence of HBV and syphilis was lower in the younger group aged 18–34 years compared to those aged 35 years and older. This pattern is consistent with age-stratified infection prevalence reported in various epidemiological studies. HIV and syphilis have been found to be more common in the 21–30 years age group, HBV in the 21–40 years age group, and HCV in the 41–60 years age group [35]. Coinfections involving HIV, HCV, or syphilis are most frequently observed in the 21–30 age range [36]. A recent study of HBV infections among Polish blood donors found that occult HBV infections increased significantly alongside donor age [37]. The relatively lower HBV prevalence in younger individuals in our study likely reflects the impact of the national HBV vaccination program initiated in August 1998 [19], which has progressively protected successive birth cohorts.

Age-related immune changes may also contribute to differential infection susceptibility, though this interpretation remains speculative. Immunosenescence, characterized by thymic involution that reduces naïve T cell output at approximately 3% per year until age 40 and 1% thereafter, along with chronic low-grade inflammation (“inflammaging”) with elevated IL-6 and TNF-α, has been associated with impaired pathogen clearance and may create 2–4 fold increases in infection susceptibility among older adults [38–40]. However, behavioral factors such as cumulative exposure time, changes in sexual practices across life stages, participation in screening programs, and differential access to healthcare services across age groups likely also play important roles in the observed age-related patterns. These findings suggest that both biological aging and social determinants contribute to the elevated TTI risk observed in older donor populations.

### Education level and prevalence of TTIs

Lower education (≤ 8 years) was associated with increased risk for all TTIs in our study. This finding aligns with previous reports indicating that lower education levels may increase seroprevalence of infections such as HIV, HBV, HCV, and syphilis among blood donors [41]. However, some studies have reported no significant relationship between education level and increased risk of certain infections such as HCV [42], suggesting that education level is a complex factor with potentially varying effects across different pathogens. Studies across 16 countries have revealed that only 28.5% of students have sufficient knowledge about blood donation, with private university students showing greater awareness [43]. In Tanzania, replacement donors from lower socioeconomic groups showed higher TTI prevalence (12.3%) than voluntary donors (9.5%) [44]. Globally, large disparities persist: only 76% of donations are adequately screened in low-income countries compared to 99.8% in high-income settings [2]. Education level likely influences infection risk through multiple pathways, including awareness of transmission routes, healthcare-seeking behavior, and socioeconomic conditions affecting exposure risk. These findings support the value of targeted educational interventions for blood donor populations, particularly those with lower educational attainment.

### Blood group associations with TTIs

Several potential mechanisms have been proposed by which blood groups might influence infection susceptibility, though our cross-sectional observational design cannot establish whether any of these mechanisms underlie our observed associations. Blood group antigens may serve as receptors or co-receptors for microorganisms, parasites, and viruses, potentially facilitating pathogen attachment to host cells and initiating infection [3]. Natural anti-A and anti-B antibodies may act as part of innate immunity, providing initial defense against pathogens expressing antigen-like structures [45]. Additionally, pathogens might employ molecular mimicry of host blood group antigens [46], which could hinder immune recognition and enable persistence or dissemination of infection [47]. These mechanisms establish a theoretical basis by which ABO and Rh blood groups might function as non-modifiable risk factors, but confirmation requires mechanistic studies beyond the scope of epidemiological observation.

Our data demonstrate that individuals with blood group B Rh(+) showed increased risk of HBV infection, whereas those with blood group A Rh(-) appeared to have a protective association. These findings are partially consistent with some individual reports describing association between blood group B and increased HBV risk [48]. The protective effect observed in A Rh(-) is partially supported by previous evidence suggesting Rh-negative individuals had a 47% lower likelihood of acquiring HBV compared to Rh-positive individuals [49]. However, our observation of increased risk in B Rh(+) individuals contrasts with the meta-analysis by Jing et al. [50], which reported blood group B was associated with an 8% lower HBV risk (RR = 0.92, 95% CI 0.86–0.98) overall.

Several methodological and population-specific factors may explain this discrepancy. First, the meta-analysis pooled 38 studies from diverse geographic regions and ethnic populations, analyzing ABO and Rh systems separately or omitting Rh analysis entirely. In contrast, our study examined combined ABO-Rh phenotypes, revealing that associations may be Rh-dependent—a nuance masked when systems are analyzed in isolation. The meta-analysis found blood group B to be protective overall, but this pooled estimate may mask important effect modification by Rh status. Second, our homogeneous Turkish population with intermediate HBV endemicity (0.594%) differs substantially from the predominantly Asian populations in the meta-analysis (where B was protective with RR = 0.91) and studies from higher HBV endemic regions (≥ 5% prevalence), where different immune selection pressures and HBV genotype distributions may drive distinct associations. Third, the meta-analysis acknowledged substantial heterogeneity (I²>50% in several subgroups) and noted limited ability to control for confounders, while our analysis adjusted for multiple covariates including sex, education, and age in multivariable models. Fourth, our blood donor cohort represents a pre-selected healthy population with lower-risk behavioral profiles, potentially revealing genetic susceptibility patterns obscured in general populations. Other studies have reported no significant ABO/Rh-HBV associations [46, 51], while findings from China suggested increased risk in blood group O [52], highlighting the heterogeneous nature of these relationships across populations.

Speculative mechanisms that might explain blood group-HBV associations warrant consideration, though they remain unconfirmed by our data. HBV utilizes sodium taurocholate co-transporting polypeptide (NTCP) as its primary entry receptor [53], indicating ABO antigens are not directly employed as viral receptors. Instead, ABO antigens might theoretically influence immunity through alternative mechanisms such as modulating cytokine levels, E-selectin expression and leukocyte adhesion to vascular walls, plasma protein interactions, or the magnitude of T-cell responses [54]. Since HBV envelope proteins (HBsAg) undergo N-glycosylation at position N146 within the immunodominant “a” determinant—a modification critical for viral infectivity and immune evasion—and the ABO gene encodes glycosyltransferase enzymes, blood group might potentially influence viral glycosylation patterns affecting immune recognition [55]. However, these remain speculative hypotheses requiring dedicated mechanistic studies.

For syphilis, we observed increased risk in B Rh(+) and AB Rh(+) individuals, with protective association in O Rh(+). These findings differ from much of the existing literature; most previous studies reported no significant ABO/Rh-syphilis associations [14, 48, 51, 56], while one study from Iraq found highest syphilis seropositivity in blood group A [57]. The protective effect observed in blood group O is partially consistent with broader observations suggesting a general protective role of this group against several diseases [58]. The unique biology of Treponema pallidum may explain the generally inconsistent literature. T. pallidum possesses an outer membrane with unusually low density of transmembrane proteins compared to other Gram-negative bacteria—a remarkable lack of antigenicity that enables immune evasion and resistance to antibody-mediated attack [59]. Consequently, mechanisms involving blood group antigens as direct pathogen receptors—often implicated in viral and bacterial infections—may not be applicable to syphilis. Our findings should therefore be interpreted cautiously as hypothesis-generating and require replication in independent cohorts.

No significant associations were found between blood groups and HCV or HIV infections in our study. The modest effect sizes observed for HBV and syphilis associations (aORs ranging from 0.855 to 1.121) underscore that while statistically significant in our large sample, these relationships have limited clinical predictive value for individual risk assessment.

### Methodological considerations

The contradictory findings regarding blood group-infection associations across the literature reflect substantial methodological heterogeneity that complicates interpretation and prevents establishment of generalizable conclusions. Blood group distributions vary across populations, as do HBV genotype distributions, which may influence disease progression differently by geography. Study populations have ranged from blood donors to general populations and patient groups, with widely varying sample sizes affecting statistical power. Diagnostic markers used to define HBV infection (HBsAg, anti-HBc, HBV DNA) differ between studies, as do syphilis screening methods (treponemal vs. non-treponemal tests), potentially influencing outcome ascertainment. Additionally, HBV vaccination programs have altered infection prevalence differentially across populations, particularly in younger cohorts. These considerations highlight the challenges of generalizing blood group-infection associations across diverse settings and underscore the need for standardized methodological approaches in future research.

In conclusion, this study demonstrates declining TTI prevalence among blood donors in Türkiye and identifies demographic risk factors including male sex, older age, and lower education that are independently associated with increased infection risk. The observed blood group associations with HBV and syphilis, while statistically significant, are modest in magnitude and should be interpreted as hypothesis-generating rather than definitive. The observed increased risk for HBV in blood group B Rh(+) and for syphilis in blood groups B Rh(+) and AB Rh(+) challenges prevailing conclusions from large meta-analyses, highlighting the population-specific nature of these associations. This complexity does not invalidate our data; rather, it underscores their value as evidence that blood group-infection relationships represent dynamic interplay between pathogen biology, host immunity, and environmental factors rather than simple genetic determinism. Future multi-center studies with standardized diagnostic criteria and comprehensive covariate assessment are needed to clarify these associations across diverse populations and to elucidate potential underlying mechanisms through dedicated molecular and genetic investigations.

## Supplementary Information

Below is the link to the electronic supplementary material.

Supplementary Material 1

Supplementary Material 2

## Data Availability

The data that support the findings of this study are available upon request from the corresponding author; however, the data are not publicly available due to privacy or ethical restrictions.

## Abbreviations

CI: Confidence Interval
FDR: False Discovery Rate
HBV: Hepatitis B Virus
HCV: Hepatitis C Virus
HIV: Human Immunodeficiency Virus
ISBT: International Society of Blood Transfusion
NAT: Nucleic Acid Amplification Testing
NTCP: Sodium Taurocholate Cotransporting Polypeptide
OR: Odds Ratio
TRC: Turkish Red Crescent
TTI: Transfusion-Transmitted Infections
WHO: World Health Organization
