# Supplementary figures and images for: Association between ABO/Rh blood groups and transfusion-transmitted infections among Turkish blood donors: a comprehensive demographic analysis (2015–2021)
Source: BMC Infect Dis. 2026 Feb 13;26:407. doi: 10.1186/s12879-026-12842-5 (PMC12917975; doi:10.1186/s12879-026-12842-5)

**Supplement figure 1.** Serological and NAT screening process.


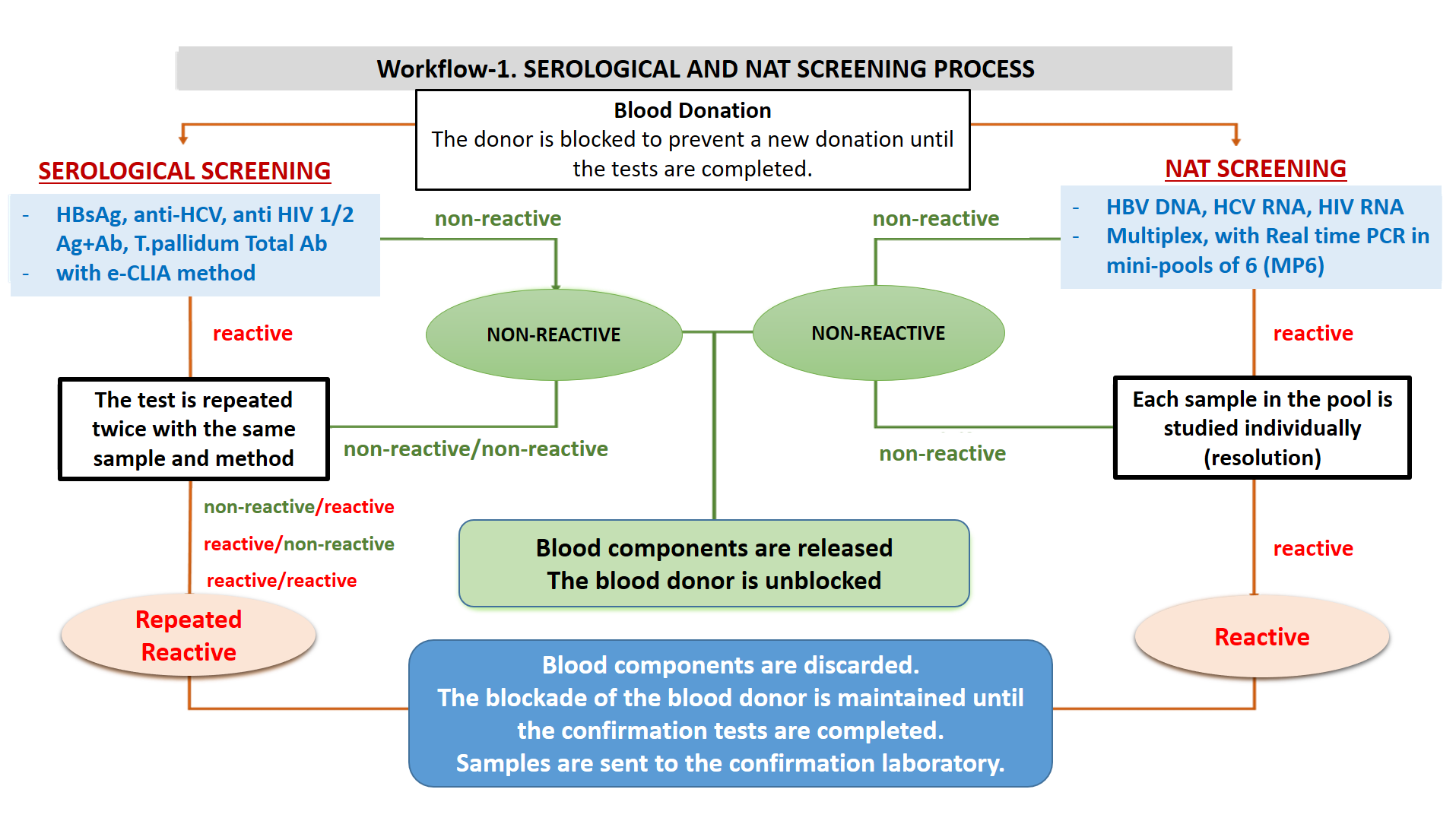

Supplement: Supplementary file 1 — Supplementary Material 1 [file 12879_2026_12842_MOESM1_ESM.docx]
