# Supplementary material for: Association between ABO/Rh blood groups and transfusion-transmitted infections among Turkish blood donors: a comprehensive demographic analysis (2015–2021)
Source: BMC Infect Dis. 2026 Feb 13;26:407. doi: 10.1186/s12879-026-12842-5 (PMC12917975; doi:10.1186/s12879-026-12842-5)
Supplement: Supplementary file 2 — Supplementary Material 2 [file 12879_2026_12842_MOESM2_ESM.docx]

***Supplement table-1:*** *Test methods and kits used in screening and confirmatory tests*

| **Test Type** | **Method** | **Kit** |
| --- | --- | --- |
| ***Serological screening*** |  |  |
| Anti-HCV | EIA | DIASORIN Murex anti-HCV (v. 4.0), *United Kingdom (2015-2019)* |
|  | CLIA | DIASORIN Liaison XL Murex HCV Ab, *Italy (2013-2019)*  ROCHE Cobas Elecsys Anti-HCV II, USA (2019 and present) |
| HBsAg | EIA | DIASORIN Murex HBsAg v.3, *United Kingdom (2015-2019)* |
|  | CLIA | DIASORIN Liaison XL Murex HBsAg Quant, *Italy (2013-2019)*  ROCHE Cobas Elecsys HBsAg II, USA (2019 and present) |
| HIV 1/2 Ag+Ab | EIA | DIASORIN Murex HIV Ag/Ab Combination, *United Kingdom (2015-2019)* |
|  | CLIA | DIASORIN Liaison XL Murex HIV Ab/Ag, *Italy (2013-2019)*  ROCHE Cobas Elecsys HIV Duo, USA (2019 and present) |
| T.pallidum Total Ab | EIA | DIASORIN ICE Syphilis, *United Kingdom (2015-2019)* |
|  | CLIA | DIASORIN Liaison Treponema Screen, *Italy (2013-2019)*  ROCHE Cobas ElecsysSyphilis, USA (2019 and present) |
| ***Serological Confirmation*** | | |
| Anti-HBc | EIA  CLIA | DIASORIN Murex anti-HBc (total), *United Kingdom (2015-2019)*  ROCHE Cobas Elecsys Anti-HBc, Germany (2019 and present) |
| HBsAg Neutralization | EIA  CLIA | DIASORIN Murex HBsAg Confirmatory V.3, *United Kingdom (2015-2019)*  ROCHE Cobas Elecsys HBsAg Confirmatory, Germany (2019 and present) |
| Anti HCV LIA | LIA | FUJIREBIO Inno-LIA HCV Score, *Belgium (2007* and present*)* |
| Anti-HIV 1-2 LIA  FTA-ABS | LIA  IIFT | FUJIREBIO Inno-LIA HIV I/II Score, *Belgium (2007* and present*)*  Euroimmun Anti-Treponema pallidum IIFT Germany (2010 and present) |
| ***NAT Screening*** | | |
| HBV DNA  HCV RNA  HIV-1 RNA  HIV-2 RNA | Real time PCR  (Multiplex,MP6) | ROCHE Cobas s-201 platform, *Switzerland (2014-2019)*  ROCHE Cobas MPX v2.0 kiti, *Switzerland (2019* and present*)* |
| ***NAT Confirmation*** | | |
| Quantitative HBV DNA | Real Time PCR | ABBOTT Real Time HBV (ABBOTT m2000sp + ABBOTT m2000rt), USA *(2015-2018)*  ROCHE Cobas HBV (ROCHE Cobas 6800), Germany *(2018* and present*)*  ROCHE Cobas Ampliprep/Cobas TaqMan HBV Test, v. 2.0, USA *(2015)* |
| Quantitative HCV RNA | Real Time PCR | ABBOTT Real Time HCV (ABBOTT m2000sp + ABBOTT m2000rt), USA *(2015-2018)*  ROCHE Cobas HCV (ROCHE Cobas 6800), Germany *(2018* and present*)*  ROCHE Cobas Ampliprep/Cobas TaqMan HCV Quantitative Test, v. 2.0, *Switzerland (2015)* |
| Quantitative HIV-1 RNA | Real Time PCR | ABBOTT Real Time HIV-1 (ABBOTT m2000sp + ABBOTT m2000rt), USA *(2015-2018)*  ROCHE Cobas HIV-1 (ROCHE Cobas 6800), Germany *(2018* and present*)*  ROCHE Cobas AmpliPrep/CobasTaqMan HIV-1 Test,  v. 2.0, USA *(2015)* |
| *(EIA; Enzyme immunoassay, CLIA; Chemiluminescence immunoassay, LIA; The Line immunoassay, NAT: Nucleic Acid Amplification Test; PCR: Polymerase Chain Reaction; MP6;MiniPool-6, Six-sample pool)* | | |
